# Association of One-Step Nucleic Acid Amplification Detected Micrometastases with Tumour Biology and Adjuvant Chemotherapy

**DOI:** 10.1155/2017/4971096

**Published:** 2017-06-12

**Authors:** Ghaleb Goussous, Sadaf Jafferbhoy, Niamh Smyth, Lisette Hammond, Sankaran Narayanan, Robert Mark Kirby, Soni Soumian

**Affiliations:** ^1^University Hospitals of North Midlands, Newcastle Road, Stoke-on-Trent, Staffordshire ST4 6QG, UK; ^2^School of Medicine, Keele University, David Weatherall Building, Stoke-on-Trent, Staffordshire ST5 5BG, UK

## Abstract

One-step nucleic acid amplification (OSNA) is an intraoperative technique with a high sensitivity and specificity for sentinel node assessment. The aim of this study was to assess the impact of OSNA on micrometastases detection rates and use of adjuvant chemotherapy. A retrospective review of patients with sentinel node micrometastases over a five-year period was carried out and a comparison of micrometastases detection using OSNA and H&E techniques was made. Out of 1285 patients who underwent sentinel node (SLN) biopsy, 76 patients had micrometastases. Using H&E staining, 36 patients were detected with SLN micrometastases (9/year) in contrast to 40 patients in the OSNA year (40/year) (*p* < 0.0001), demonstrating a fourfold increase with the use of OSNA. In the OSNA group, there was also a proportional increase in Grade III, triple-negative, ER-negative, and HER-2-positive tumours being diagnosed with micrometastases. Also on interactive PREDICT tool, the number of patients with a predicted 10-year survival benefit of more than 3% with adjuvant chemotherapy increased from 52 to 70 percent. OSNA has resulted in an increased detection rate of micrometastases especially in patients with aggressive tumour biology. This increased the number of patients who had a predicted survival benefit from adjuvant chemotherapy.

## 1. Introduction

Axillary lymph node status at the time of initial diagnosis is one of the most important prognostic indicators for breast cancer. The goal of axillary node clearance (ANC) is to provide accurate staging information and local control of the disease. However, it has many potential complications including lymphedema, seroma, shoulder dysfunction, and paraesthesia. Sentinel lymph node (SLN) biopsy has replaced ANC due to its lower morbidity and equivalent long-term prognosis [1, 2]. Several methods of SLN assessment exist ranging from the conventional gold-standard haematoxylin and eosin (H&E) histopathological examination with or without immunohistochemistry staining to more rapid intraoperative assessment techniques that offer the ability to make a decision about axillary surgery at the time of the primary procedure.

The one-step nucleic acid amplification (OSNA) is an intraoperative technique which detects and quantifies the presence of cytokeratin 19 (CK-19) mRNA in a lymph node by polymerase chain reaction (PCR) [3]. This technology has been validated by several authors [4–13] and endorsed by National Institute of Clinical Excellence in August 2013 [14]. There are suggestions that OSNA has resulted in increased detection of metastases, especially micrometastases, in comparison to traditional methods [15–17]. However, the management of axilla with sentinel node micrometastases has changed over the last few years. ANC was routinely carried out in patients with sentinel node micrometastases but studies have shown no survival advantage [18]. There is also evidence to suggest that micrometastases in itself should not be an indication for systemic treatment [19]. Since the introduction of OSNA in our institution in November 2012, we have observed a trend towards increased detection of SLN micrometastases with a potential impact on adjuvant treatment decisions. Therefore we assessed the rates of micrometastases diagnosed by OSNA and the use of adjuvant chemotherapy.

## 2. Materials and Methods

This study was carried out in a University Hospital which deals with around 600 breast cancer referrals per annum. All patients who underwent SLN biopsy between January 2009 and December 2013 were included. Retrospective data on demographic details, presentation, type of surgery, number and status of lymph nodes, and histological tumour details was collected and analysed.

SLN biopsy was performed using a conventional dual technique of intradermal technetium 99 radioactive nanocolloid injection and subdermal blue dye injection (patent blue). Before November 2012, histological evaluation was carried out using standard H&E staining. All sentinel nodes were sent as formalin fixed specimen to the lab. The nodes were the sectioned in 2.0 mm slices before embedding them in paraffin blocks. H&E staining was only carried out in the sentinel node assessment. In November 2012, OSNA was adopted in our institution and underwent a 3-month period of internal validation where half the SLN was examined by OSNA and the other half was sent for traditional assessment. We adopted the standard CK-19 mRNA copy count to stratify the size of metastasis where mRNA copy count of <250 copies/*μ*L, 250–5000 copies/*μ*L, and >5000 copies/*μ*L is considered as negative, micrometastases, and macrometastases, respectively [3].

The mean sentinel node retrieval rate was two, both in pre- and post-OSNA group. ANC was performed if one or more SLNs were positive for macrometastases. The management of micrometastases changed during the course of use of OSNA; in the beginning of the study period the surgeons were more inclined to carry out ANC if micrometastases were found but in the latter part, a more judicious approach was adopted based on the change in attitude towards ANC. The data was collated and analysed using Microsoft Excel (Microsoft Corporation, Redmond, USA). *p* values were calculated using the Chi-square test (*χ*^2^). Ten-year survival benefit with adjuvant chemotherapy was retrospectively calculated using PREDICT online, version 1.2 [20], where micrometastases were considered node negative for calculation purposes.

## 3. Results

In the 5-year study period between January 2009 and December 2013, a total of 1285 SLN biopsies were performed out of which 76 patients had SLN micrometastases (6%). The demographic details and tumour characteristics are given in Table 1.

**Figure 1:**
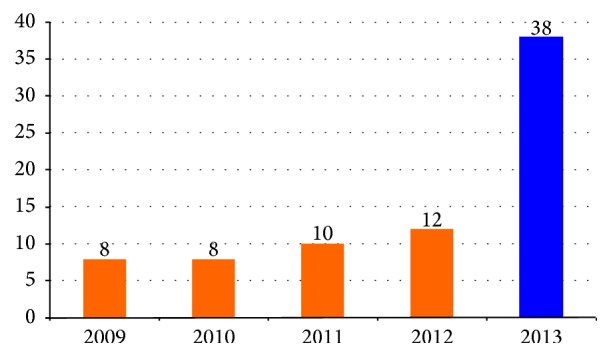
Distribution of detected micrometastases over the study period (*p* < 0.0001).

In the pre-OSNA period of the study (January 2009–November 2012), 36 cases with micrometastases were detected with a consistent detection rate of 9 cases per annum. There was a significant increase in detection of micrometastases with the introduction of OSNA (*p* < 0.0001). This is despite a steady caseload of cancers per annum (Figure 1).

**Figure 2:**
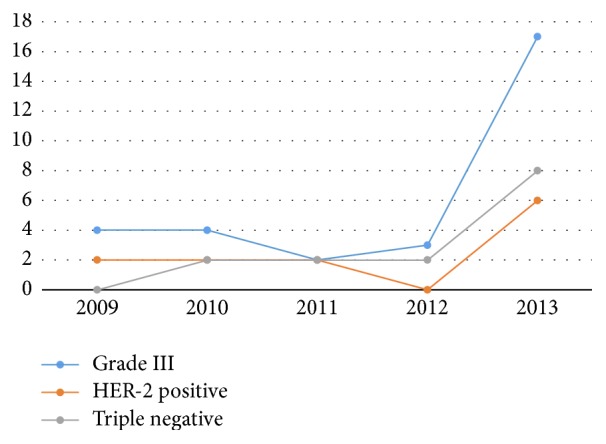
Distribution of micrometastases in aggressive tumours during the study period.

After the introduction of OSNA, a fourfold increase in Grade III tumours with micrometastases was noted. A similar trend was noted in triple-negative cases and the number of HER-2-positive cases increased from 2/annum to 6 in the OSNA year (Figure 2) and ER-negative cases increased from 14.1% to 25% (Table 1).

In the pre-OSNA period 24 symptomatic (66.7%) and 12 screen-detected (33.3%) patients were found to have micrometastases. In the post-OSNA period, however 19 symptomatic (47.5%) and 21 screen-detected (52.5%) patients had micrometastases detected (NS).

During this study period, 34 out of 76 patients (44.7%) over the study period underwent ANC on the basis of micrometastases detection. In the pre-OSNA period, 26 patients (72.2%) underwent ANC and 8 patients (30.7%) were found to have further positive nonsentinel axillary nodes. In contrast, out of 8 patients (20%) who underwent ANC in the OSNA period, only one (12.5%) was found to have further positive nonsentinel nodes (NS).

**Figure 3:**
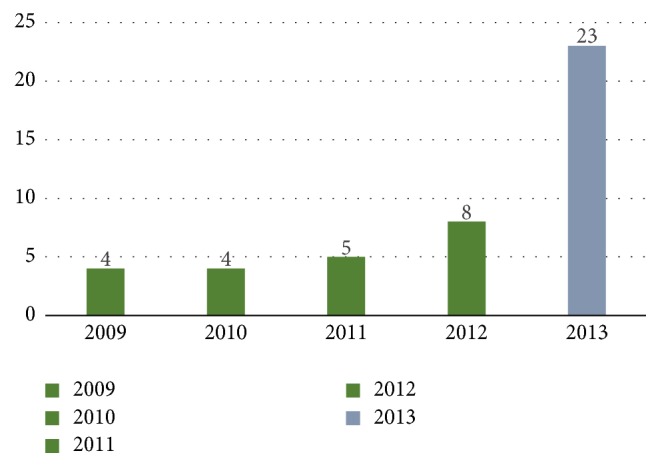
Adjuvant chemotherapy before and after the introduction of OSNA.

Twenty-one patients (58.3%) in the pre-OSNA period and 23 patients (57.5%) in the OSNA period received adjuvant chemotherapy (Figure 3). The tumour characteristics of these patients are given in Table 2. Eight patients in the pre-OSNA group and 2 patients in the post-OSNA group with aggressive tumour biology (Grade III, triple-negative, or HER-2-positive) did not receive chemotherapy due to factors such as age, comorbidities, or patients' choice.

Using the PREDICT tool, a survival advantage from chemotherapy in both of these groups was calculated. In the chemotherapy group, 11 patients (52%) in the pre-OSNA period and 16 patients (70%) in the OSNA period had a predicted 10-year survival benefit of >3% with systemic treatment.

## 4. Discussion

Our study has demonstrated a fourfold increase in micrometastases detection rate with the introduction of OSNA. In the last year of the study, when OSNA was adopted, almost as many micrometastases were detected as in the previous four years. The reason for this discrepancy is not entirely clear. A possible explanation could be that the whole node is used for OSNA analysis rather than only sections. It could also be due to human factors and varying interpretation of histological specimens and technique of node processing itself. Turner and his colleagues highlighted interobserver discrepancies when they demonstrated that the interpretations of six experienced breast pathologists varied in 23.8% when asked to review 56 electronic images of small-volume disease [21]. Cserni et al. showed that different interpretations of the TNM definitions could cause variations in the classification of small-volume disease and therefore impact the staging of the disease [22]. Conventional histopathology may underestimate the size of the metastasis leading to possible false-negative results. Intensive examination of the node is essential to pick up low-volume deposits [23] with section thickness of about 200–250 *μ*m needed to demonstrate micrometastases reliably [24]. This can increase pathology workload enormously and cannot be done intraoperatively. We believe that OSNA analysis of the node in its entirety detects deposits that would have otherwise been missed by the pathologist. In our study, the 4-fold increase in the detection of micrometastases could have been due to the same reason. These views were corroborated in a study by Osako et al. who whilst finding no difference in detecting macrometastases between OSNA and frozen section found that OSNA detected significantly more micrometastases [15]. A plausible explanation for the increased detection rates with OSNA is false-positive results due to potential contamination with benign epithelial cells [25]. Another possibility is that considering the sensitivity of the technique, OSNA could be picking up Isolated Tumour Cells and interpreting them as micrometastases. In this context, a recent meta-analysis has commented on the potential high false positives with the technique of OSNA [17].

In our study, we observed that the micrometastases detection rate in the screening population rose considerably after the introduction of OSNA (33.3% to 52.5%). It is possible that screening patients with early disease could have had smaller SLN deposits that would have otherwise been missed on histopathology. Unsurprisingly the screening population in our cohort had smaller average tumour size than the symptomatic population (19.6 mm versus 26.3 mm) as reported in literature [26–28].

Further nonsentinel node positivity following ANC in patients with micrometastases was higher in the pre-OSNA group (30.7% versus 12.5%). This finding is possibly a reflection of the sensitivity of OSNA or the potential for slightly increased false-positive results with its use. However, the numbers in our series are too low to make any practical interpretation.

The prognostic significance and therapeutic implications of SLN micrometastases remain a matter of great debate. With the use of OSNA, the information provided is difficult to apply in clinical setting. Studies have shown that SLN micrometastases are not associated with worse prognosis than node-negative patients [29]. Therefore, chemotherapy should not be recommended to all patients particularly the ones who do not have aggressive tumour characteristics as the potential benefit from chemotherapy is small and side-effects are considerable. In our study, there was an increase in the number of OSNA detected micrometastases in HER-2-positive cases as well as ER-negative cases which can only be explained by the ability of OSNA to detect micrometastases in patients with more aggressive biology. With the increase in the rate of micrometastases, there was also an increase in the number of patients who had a predicted survival advantage with chemotherapy in OSNA period.

In our practice, PREDICT online is routinely used to assess long-term prognosis in order to decide about adjuvant treatment options. It takes into account the mode of presentation, size, and grade of tumour along with the estrogen and HER-2 receptor status. The predictive accuracy of this online tool has been validated by many studies published in literature [30, 31]. Patients with a predicted survival advantage of >3% are offered adjuvant chemotherapy. On the PREDICT score, 11% patients in the pre-OSNA period had a 10-year survival advantage of >3% in contrast to 16% patients in the post-OSNA group. This could be due to the proportionally higher number of patients with Grade III, triple-negative, and HER-2-positive tumours in the OSNA period. It could be argued that the increase in the micrometastases detection rate during the OSNA period may be due to the biological features of the tumour with the sensitive OSNA technique picking up axillary disease in aggressive variants of cancer.

A recent systematic review on the clinical effectiveness of OSNA concluded that a lack of histological information such as the location of metastatic foci and the morphological features with intraoperative analysis reduces the diagnostic accuracy. This has also raised the question if the cost reduction with intraoperative testing is worth the reduction in diagnostic accuracy and potentially inferior long-term health outcomes for patients [32]. An interesting finding in our study was a fourfold increase in detection of micrometastases in aggressive tumour variants with the use of OSNA. These micrometastases were probably missed in the pre-OSNA era and in the context of having poor prognostic tumour are likely to be true metastatic deposits. The increase in micrometastases detection rate in the OSNA year reflects the sensitivity of this technique.

## 5. Conclusion

OSNA has resulted in increased rate of detection of micrometastases. There was also an increased detection rate of micrometastases particularly in patients with aggressive tumour characteristics and in the use of adjuvant chemotherapy, but this was more likely to be due to the tumour biology.

## Figures and Tables

**Table 1:** Demographic and tumour characteristics.

	Pre-OSNA	Post-OSNA
	(2009–2012)	(2013)
*Number of patients (n/%)*	36 (47.4%)	40 (52.6%)
*Age (range)*	57 (39–74)	60 (32–76)
*Screen detected*	12 (33.3%)	21 (52.5%)
*Tumour size (mm)*	25.9	18.3
*Tumour grade (n/%)*		
Grade 1	5 (14%)	2 (5%)
Grade 2	18 (50%)	21 (52.5%)
Grade 3	13 (36%)	17 (42.5%)
*ER receptor (n/%)*		
Positive	31 (86%)	28 (70%)
Negative	5 (14%)	11 (27.5%)
Borderline	0	1 (2.5%)
*HER-2 receptor (n/%)*		
Positive	6 (16.6%)	6 (15%)
Negative	30 (83.3%)	34 (85%)
*Triple negative (n/%)*	6 (16.6%)	8 (20%)

**Table 2:** Tumour characteristics of patients who underwent adjuvant chemotherapy.

	Pre-OSNA	Post-OSNA
	(2009–2012)	(2013)
*Number of patients (n/%)*	21	23
*Age (range)*	54 (39–69)	53 (32–74)
*Screen detected*	6 (28.5%)	6 (26%)
*Tumour size (mm)*	26.5	21.8
*Tumour grade (n/%)*		
Grade 1	1 (5%)	0 (0%)
Grade 2	12 (57%)	7 (30%)
Grade 3	8 (38%)	16 (70%)
*ER receptor (n/%)*		
Positive	17 (80.9%)	14 (60.8%)
Negative	4 (19.1%)	8 (38%)
Borderline	0	1 (1.2%)
*HER-2 receptor (n/%)*		
Positive	2 (9.5%)	6 (25.1%)
Negative	19 (90.5%)	17 (73.9%)
*Triple negative (n/%)*	4 (19%)	6 (26%)
*Predicted 10-year survival benefit with chemotherapy >3%*	11 (52%)	16 (70%)
